# Ethnoveterinary practises of medicinal plants used for the treatment of different cattle diseases: A case study in East Khasi Hill district of Meghalaya, North East India

**DOI:** 10.1016/j.heliyon.2023.e18214

**Published:** 2023-07-13

**Authors:** Nazir Ahmad Bhat, Licha Jeri, Dolly Karmakar, Puranjoy Mipun, Pankaj Bharali, Nilofer Sheikh, Chester John Nongkynrih, Yogendra Kumar

**Affiliations:** a Centre for Advanced Studies in Botany, North-Eastern Hill University, Shillong, 793022, Meghalaya, India; b Department of Botany, University of Science and Technology (USTM), Ri-Bhoi, 793101, Meghalaya, India; c Department of Botany, Bhattadev University, Bajali, 781325, Assam, India; d Centre for Infectious Diseases, Biological Sciences and Technology Division (BSTD), CSIR-North East Institute of Science and Technology, Jorhat, 785006, Assam, India; e Department of Botany, Biswanath College, Biswanath Chariali, 784176, Assam, India

**Keywords:** Bioactive compounds, Cattle, Diseases, Ethnomedicine, Indigenous knowledge, Phytochemicals, Traditional healers

## Abstract

**Introduction:**

For generations, the inhabitants of Meghalaya have relied on medicinal plants to maintain the health of their livestock and treat various illnesses that may afflict their animals. Due to the lack of survey for use and documentation, these plants have never been undertaken. Therefore, it is imperative to explore the diversity, utilization, and phytochemical profile of these plants and quantitatively analyse the data to identify important medicinal plants. By doing so, we can better understand the potential of these plants for developing novel drugs.

**Methods:**

Frequent field trips were made for the collection of ethnoveterinary data of medicinal plants from local animal-keepers, traditional healers (THs) and inhabitants of different age groups. This information was gathered through semi-structured interviews, individual discussions, direct field-use observation, and questionnaires. A total of 52 informants (35 females and 17 males) were interviewed from seven rural villages and the information obtained from them were quantitatively analysed using the informant consensus factor (ICF), and fidelity level (FL). Additionally, for each documented plant, available published literature was extensively surveyed to identify the presence of bioactive chemical compounds responsible for their therapeutic effects.

**Results:**

During the present study, a total 96 plants, distributed into 87 genera and 43 families were identified and recorded for their use in ethnoveterinary practices against more than 25 diseases. Out of the recorded plant species, the Fabaceae family was found to be the most dominant with seven species, followed by Poaceae and Lamiaceae with six species each, and Moraceae with five species. The leaves (50.00%) and seeds (12.50%) were the most frequently used plant parts, while the paste (30 species) was the common mode of application. *Aegle marmelos* Correa exhibited a fidelity level (FL) of 100% for indigestion, while *Tagetes erecta* L. had a fidelity level of 94.11% for wound treatment, making them the most promising candidates for further study. The highest FIC value of 1.00 was recorded for the treatment of neurological disorder (1.00), followed by foot and mouth disease (FIC 0.91), which depicted that some species were frequently utilized to treat multiple livestock ailments.

**Conclusion:**

The study presents trustworthy information about medicinal plants and their associated indigenous ethnoveterinary knowledge. It has been scientifically proven that these plants contain bioactive compounds responsible for their therapeutic properties. However, this knowledge is in danger of being lost due to factors like socioeconomic changes, environmental and technological alterations, and lack of interest from younger generations. Therefore, it is essential to document this empirical folklore knowledge systematically and take measures to protect and conserve it.

## Graphical abstract

**Figure:**
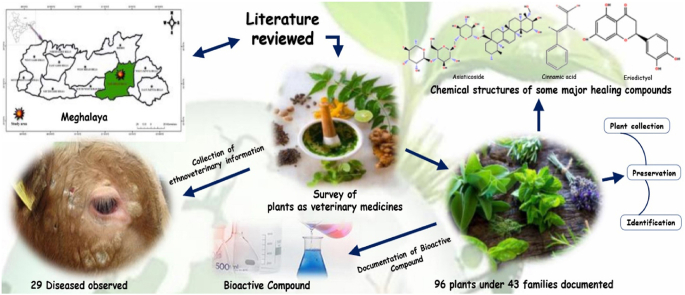

## 1 Introduction

Ethnic and religious beliefs, familiarity, expertise, methods and practices about the health of animals is ethnoveterinary medicine (EVM), which plays an important role in rural areas as a chief source of medicine to cure their livestock [1]. Ethnoveterinary knowledge (EVK) of plants is still regarded as a valuable resource that captures people's whole dedication to and experience with life, from their earliest days through all phases of evolution to the present. Throughout the world, all conventional healing practices were directly derived from plants, which play an essential role in treating and preventing a variety of diseases by local inhabitants [2,3]. It is estimated that approximately 50% of the modern drugs used today have been derived from natural products, with the majority of those derived from plants [4,5]. However, the percentage of drugs that are still obtained directly from plants has decreased over time due to advances in synthetic chemistry and drug development. Nevertheless, plants continue to be an important source of inspiration and compounds for drug discovery and development [6]. India is home to a vast collection of ethnobotanical knowledge from ancient times, which may be quite helpful in identifying the plants that were utilized in early civilisation [7]. Since that time, subsequent generations have been imprinted with that knowledge, belief and practises related to the diagnosis, treatment, and management of animal and human illnesses.

Livestock healers have vast knowledge about their animals [8], and their knowledge and skill are communicated orally from generation to generation, which is kept under the custody of old people in the rural and tribal belts throughout the world, depending on the community, ethnicity, sex, age and caste [9,10]. For the prevention of animal diseases, numerous studies have been carried out in different parts of the world, confirming that plants are regularly used as a remedy for their treatment [[11], [12], [13], [14]]. In many rural and peri-urban communities, considerable proportion of useful ethnoknowledge of animal health care practices remain unknown to date, despite the fact that they are more frequently demanded to be included in primary animal health care delivery systems for widespread usage [15]. This irreplaceable knowledge may vanish as of rapid socioeconomic, environmental and technological alterations and as a result, we lost our cultural heritage under the guise of civilization [16]. To conserve these practices, documentation through proper systematic studies is imperative before it is lost forever while carrying out scientific evaluation, modification and optimization of these traditional practices. Promoting the conservation strategies of use of ethnoveterinary medicine does not mean to undermining or disregarding the significance of modern medical care or aiming to replace it with other. However, it does mean that each form has advantages and disadvantages. They work well together in certain situations, but in others, traditional practice will be preferable, and in yet other situations, modern approaches ought to be advised.

Livestock plays an important role in the livelihood improvement and build diverse benefactions to the inhabitants of Meghalaya and are used in both economic and social activities, such as trade, human consumption (wedding, funeral, and religious functions), ploughing and manure accumulation. As per the 20th livestock census- 2019 data from the Government of India (GOI), the total cattle population of India is estimated to grow from 190.90 to 193.46 million heads, increased by 1.34% since 2012 [17]. The state of Meghalaya had 0.41% of cattle and 0.02% of Buffalo's population of the country, an overall increase of 1.6% in total cattle population during the inter-censual period [17,18].

In recent years significant progress has been made on the documentation of medicinal plants used traditionally to cure animal diseases by ethnic communities of different parts of India [14,[19], [20], [21], [22]] and in Northeast [[23], [24], [25], [26]], but in Meghalaya, very little attention has been given to the use and documentation of plants as veterinary medicines, as it is one of the major tribal populated State of the country. Indigenous inhabitants of the state have immense and inimitable traditional knowledge about the use of natural resources for their livestock production and management. Although in the advancement of orthodox medicine, they still believe and depend on traditional healing practices for their daily healthcare needs. Therefore, it is desirable to conserve this precious unrivalled folklore knowledge of East Khasi Hills District, Meghalaya. An attempt was made to expose the uses of plants as veterinary medicines in the unexplored region of the study area. The core aim of the present study was to document the reliable information about ethnoveterinary knowledge of traditional healers and to serve as baseline information for future chemical and pharmacological studies after proper verification for the advancement and enhancement in the animal drugs system.

## 2 Materials and methods

### 2.1 Study area

**Fig. 1:**
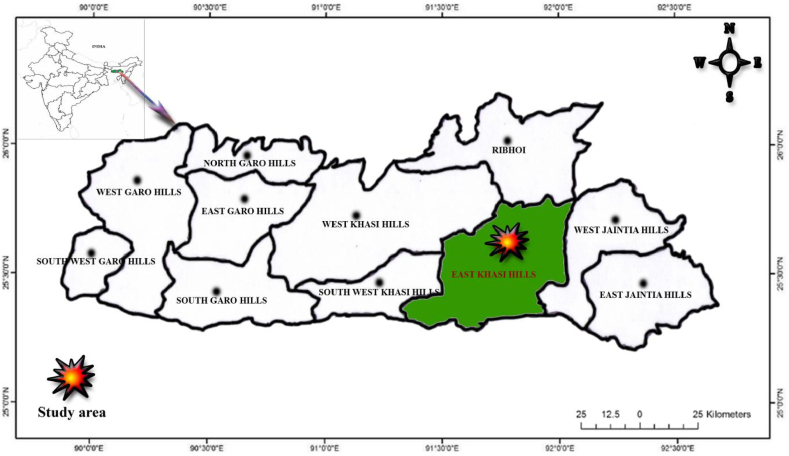
Map of the study site.

East Khasi Hill district of Meghalaya covers an area of nearly 2748 sq km^2^ and lies between 25°07″ & 25°41″ N Latitude and 91°21″ & 92°09″ E Longitude. It is located in the extreme northeastern region of the country, along with the international border with Bangladesh (Fig. 1). The altitude ranges from 150–2000 m asl, having the highest being Shillong peak point (1966 m). The climate of the study area ranges from temperate in the plateau region to the warmer tropical and sub-tropical pockets in the Northern and Southern regions. The area has highly diverse community forests as compared to the other districts of the state and they are protecting their forest by themselves.

**Fig. 2:**
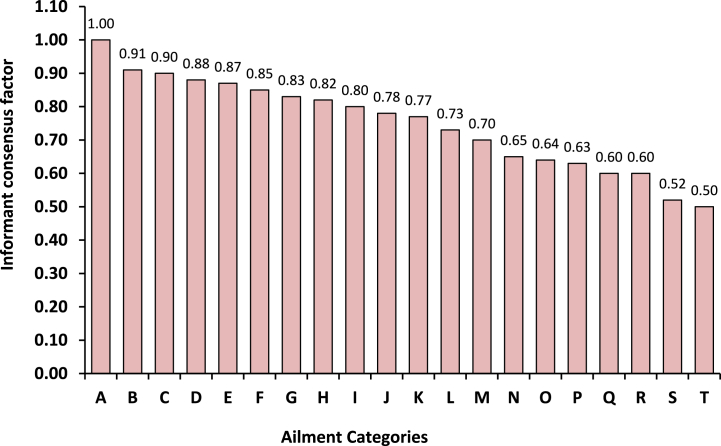
Informant consensus factor for each use category. A - Neurological disorder; B - Foot and mouth disease; C - Deworming; D - Dog, snake and insect bite; E - Cold and cough; F - Increase lactation; G - Wound treatment; H - Bone fracture and joint pain; I - Mastitis; J - Health tonic; K - Conjunctivitis; L - Diarrhoea and dysentery; M - Constipation and bloat; N - Indigestion; O - Dysuria; P - Food poisoning; Q - Skin Infection; R - Anthrax and haemorrhagic septicaemia; S - Fever and T - respiratory diseases.

### 2.2 Identification of informants

The present study attempts to survey medicinal plants used in ethnoveterinary practices. Information about their uses was collected from seven rural (hilly) village areas: Iew Pynursla, Wahlyngkhat, Umkor, Pynursla, Laitlyting, Lait Mynrieng, and Urksew Wahpathaw (Table 1). Prior to conducting the fieldwork, a meeting was arranged with the village heads to discuss the objectives and methodology of the research. In every village, resource persons or traditional healers were identified to provide ethnoveterinary information. A total of 52 practitioners aged between 35 and 60 years were identified for the survey. Periodic field surveys were carried out at the study site between November 2018 and March 2020 to collect plants and associated ethnoveterinary information.

**Table 1:** Details of surveyed villages, to identify the ethnoveterinary plants used by the local people in the East Khasi Hills District, Meghalaya, India.

Name of village	Population	Latitude (N)	Longitude (E)	Altitude (m)
**Iew Pynursla**	377	25°18′37.1″N	91°53′56.8″E	1345
**Wahlyngkhat**	813	25°19′56.8″N	91°53′34.4″E	1473
**Umkor**	1430	25°18′48.1″N	91°53′40.1″E	1373
**Pynursla**	1014	25°18′35.6″N	91°54′08.3″E	1336
**Laitlyting**	1145	25°21′09.8″N	91°53′45.5″E	1584
**Urksew Wahpathaw**	805	25°18′10.2″N	91°54′48.4″E	1290
**Lait Mynrieng**	207	25°19′24.0″N	91°53′32.9″E	1437

### 2.3 Data collection

Information about the use of plants was collected from 52 selected informants (35 female and 17 male) having traditional knowledge on folk veterinary medicines by following questionnaire survey, semi-structured interviews, individual discussions, and direct field-use observation, living in the investigated area. The informants were selected through purposive sampling based on their experience and expertise in ethnoveterinary practices. The questions were designed to elicit information on: (1) the personal characterization of participants; (2) the types of medicinal plants used in ethnoveterinary practices; and (3) the mode of preparation and administration, and the effectiveness of the treatments. A field data sheet has been prepared to record the details of plants with ethnoveterinary uses from the informants. Information viz., local name of the plant, part used for curing, method of preparation, any other plants/agents used as ingredients, modes of administration, dosage were recorded for each collected taxon. Participants interviewed in this study have given their full consent to participate and affirm that the information they have provided is true, accurate, and complete. The information was cross-checked by the information provided by the other tribal practitioners during the earlier visits.

### 2.4 Plant collection and identification

The plants mentioned by the informants were collected and identified with the help of taxonomic experts, available published literature [[27], [28], [29], [30]], botanical keys, online efloras (Flora of China, India, Nepal, Pakistan, etc.), consulting the Herbaria of Department of Botany, NEHU, Shillong and verified using World Flora Online (http://www.worldfloraonline.org/database). The voucher specimens were then catalogued and preserved in the herbarium of the Department of Botany, NEHU for future references.

### 2.5 Data analysis and statistical evaluation

Data collected from the field through interviews of the informant were tabulated in Microsoft Excel spreadsheets and analysed using two quantitative indices: informant consensus (FIC) and fidelity level (FL). Informant consensus factor (FIC) was used to find the level of homogeneity among the information provided by different informants. The informant’s consensus factor (FIC) [31] for the use of plants in the treatment of different ailments of cattle was calculated by using the following formula:

FIC = Nur − Nt/(Nur − 1)

Where Nur is the number of use reports from informants for a particular plant use category and Nt is the number of species that are used for that plant use category for all informants. FIC values range between 0 and 1, where ‘1’ indicates the highest level of informant consent.

The Fidelity level (FL %) enumerates the significance of a species for a given purpose, was calculated by using the following formula:

FL (%) = (Np/N) × 100

where, Np = number of informants who cited the species for the particular use; N = total number of informants that mentioned the plant for any use.

### 2.6 Medicinal efficacy

To determine the efficacy of documented medicinal plants used in ethnoveterinary practices, a literature survey was conducted to identify the presence of bioactive chemical compounds responsible for their therapeutic effects. Pharmacological activities of identified compounds were searched using various scientific databases, such as Google Scholar, Science Direct, PubMed, SciFinder, Web of Science, ACS Publications, and Wiley Online library by using different keywords. Therapeutic potential of the major bioactive compounds was also done by reviewing the literature that investigated the pharmacological activities of the compounds, including their antidiarrheal, antimicrobial, anti-inflammatory, antioxidant, and other bioactivities. The chemical structures of the important phytochemical compounds were drawn using ChemDraw Pro software and confirmed by PubChem (https://pubchem.ncbi.nlm.nih.gov/) database.

## 3 Results

### 3.1 Population structure of the informants

Data on ethnoveterinary practices were gathered from a diverse group of informants with varying ethnic and educational backgrounds, through a series of 52 systematic interviews. The majority of whom were analphabetic, and were middle-aged or older men (35–60 years). Out of the 52 participants, 35 were female and 17 were male. They represented various occupations, with small-scale farmers and traditional healers being the most commonly represented.

### 3.2 Documentation of traditional ethnoveterinary knowledge

**Fig. 3:**
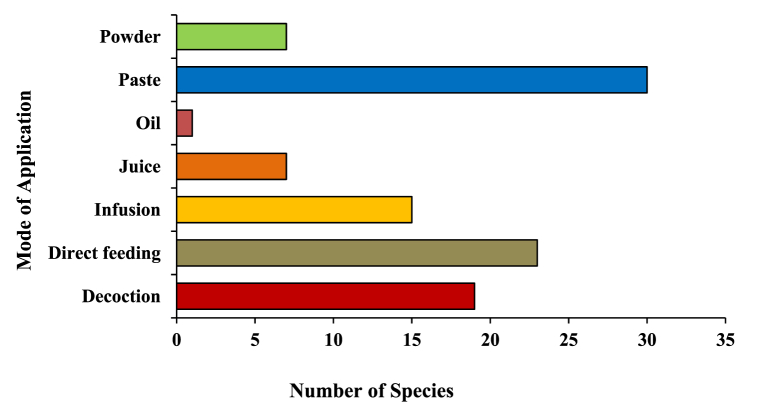
Mode of application of ethnoveterinary plant used for curing various ailments.

**Fig. 4:**
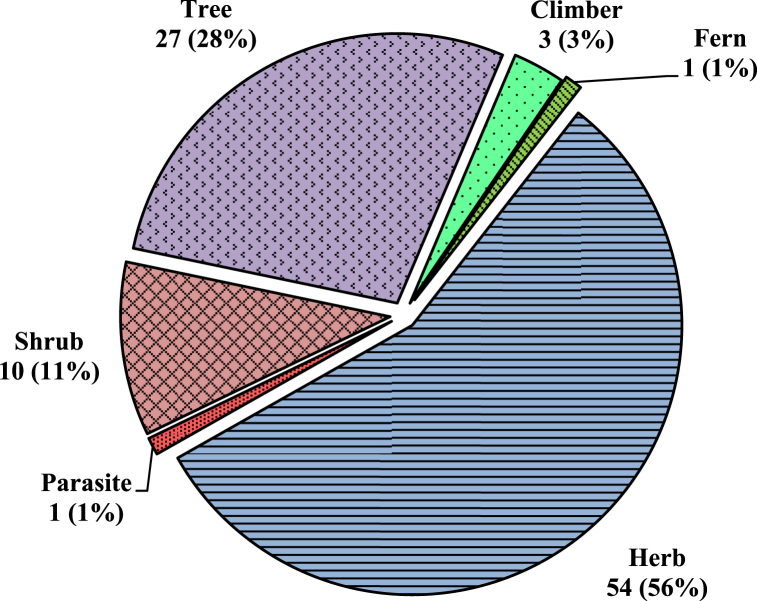
Life forms of collected ethnoveterinary plants with respect to number of species.

**Fig. 5:**
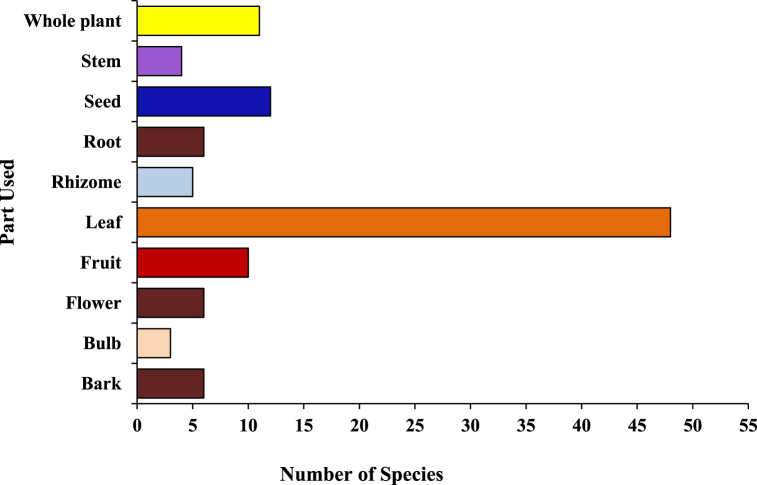
Percent distribution of plant parts used as ethnoveterinary medicine.

**Fig. 6:**
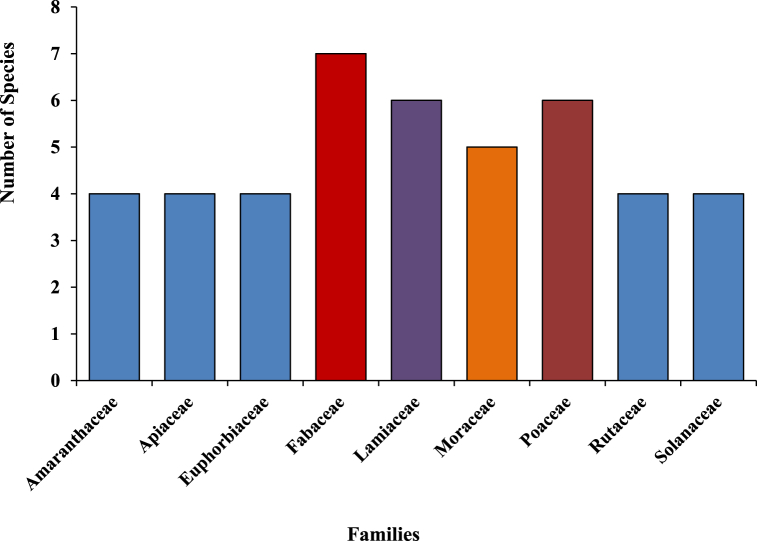
Dominant families (≥4 sp.) and their respective number of species.

**Image 1:**
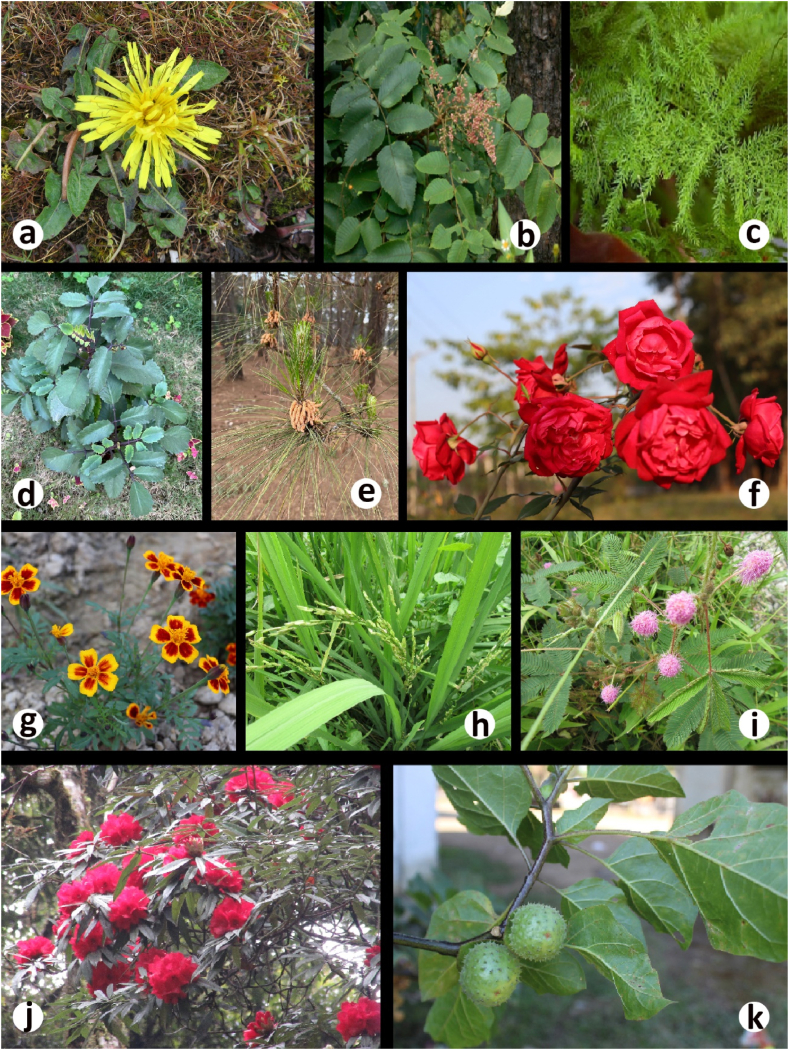
**a.** = *Taraxacum officinale* Wigg.; **b.** = *Rhus javanica* L.; **c.** = *Asparagus racemosus* Willd.; **d.** = *Bryophyllum pinnatum* (Lam.) Oken; **e.** = *Pinus kesiya* Royle ex. Gordon; **f.** = *Rosa indica* L.; **g.** = *Tagetes erecta* L.; **h.** = *Oryza sativa* L.; **i.** = *Mimosa pudica* L.; **j.** = *Rhododendron arboreum* Sm. and **k.** = *Datura metal* L.

A total of 96 plant species of different plant groups (i.e., 54 herbs, 27 trees, 10 shrubs, 3 climbers, 1 fern and 1 parasite) belonging to 43 families have been documented for the treatment of more than 20 categories of ailments (Fig. 4). For each species botanical name, voucher specimen number, local name, family, part(s) used, ailments to be treated, mode of use and mode of application was recorded and arranged family-wise in alphabetical order (Table 2). Majority of them are collected from wild (78%), while some species (21%) are in cultivation, either in gardens or fields (Image 1). Among all the plant families recorded, the most species-rich were the Fabaceae accounted 7 species, followed by Poaceae and Lamiaceae (6 spp. each) and Moraceae (5 spp.), whereas Solanaceae, Rutaceae, Euphorbiaceae, Apiaceae and Amaranthaceae contributed 4 species each (Fig. 6). Five families (Acanthaceae, Amaryllidaceae, Asteraceae, Rosaceae and Zingiberaceae) represented 3 species each and the remaining 29 reported families were constituted by only one or two species. Leaves (50.00%) were found to be the most frequently utilized plant parts even solely or mixed with other parts for ethnoveterinary usage followed by seed (12.50%), fruit (10,42%) and whole plant, while bulb (3.13%) found to be the less utilized plant parts (Fig. 5). During the treatment of illnesses, several methods of medicinal preparations were used. The most regularly used mode of preparation was paste, which accounted the highest (31.25%) followed by direct feeding (23.96%), decoction (19.79%), infusion (15.63%) juice and powder (7.29% each) while oil (1.04%) was reported in only one species (Fig. 3). In the present survey, it was recorded that the majority of preparations were taken orally (57.29%) to treat cattle ailment followed by topical routes (26.04%) and the rest 16.66% have both applications. Overall, a total of 29 sicknesses were documented during our period of the survey. Indigestion, foot and mouth disease, fever, ecto and endo parasites, deworming, skin infection, dysuria and conjunctivitis were the most frequently reported health problems (Table 2). From all the ailments occurring in the district, a total of twenty diseases were categorized.

**Table 2:** Ethnoveterinary medicinal plants used by the indigenous people for their livestock healthcare in the East Khasi Hills District, Meghalaya, India.

Family/Scientific name/voucher specimen	Vernacular name	Habit	Life cycle	Parts used	Ailments	Used type	Mode of application	Dosage forms
**Acanthaceae**								
*Andrographis paniculata* (Burm.f.) Wall. Ex Nees [NEHU-10862]	Chirota (Kh), Chirota (B)	H	PR	WP	Dysuria	O	Infusion	1–2 *l* twice a week given directly in morning hours
*Justicia adhatoda* L. [NEHU-10863]	Tdong Ksaw (Kh), Bashok pata (B)	S	PR	LF	Respiratory disease, fever, diarrhoea, dysentery	O	Paste	Dried 500 *g* mixed with jiggery and salt, given twice a day
*Peristrophe bivalvis* Merr. [NEHU-10864]	–	H	PR	LF	Health tonic	O	Direct feeding	NA
**Acoraceae**								
*Acorus calamus* L. [NEHU-10874]	Bet (Kh)	H	PR	RH	Ectoparasite control, wound treatment, indigestion	T, O	Decoction	1–2 *l* given once a day directly with water for two days
**Amaranthaceae**								
*Achyranthus aspera* (Lam.) Griseb. [NEHU-10865]	Uptooni gaach (B)	H	AN	LF	Wound treatment, after parturition, respiratory disease	T, O	Paste	NA
*Amaranthus viridis* L. [NEHU-10866]	Jada (Kh), Daata saagh (B)	H	AN	WP	Health tonic, wound treatment, increase lactation	O	Direct feeding	3–5 kg of whole plant is crushed and mixed with wheat husk for daily basis
*Alternanthera philoxeroides* (Mart.) Griseb. [NEHU-10867]	Ellencha (B)	H	AN	WP	Dysentery, diarrhoea	O	Direct feeding	3–5 kg every day
*Chenopodium album* L. [NEHU-10868]	Bathu saagh (B)	H	AN	LF, SE	Increase lactation	O	Direct feeding	NA
**Amaryllidaceae**								
*Allium cepa* L. [NEHU-10859]	Piat (Kh), Piyaj (B)	H	AN	BL	Cough, dog and insect bite	T, O	Paste	1 kg mixed with black paper powder (10 *g*) and green chillies (10 g) and water, given twice a day for cough
*Allium sativum* L. [NEHU-10860]	Rynsun (Kh), Roshun (B)	H	AN	BL	Indigestion, bloat	O	Paste	150 *g* mixed with sugar and water given twice a day
*Crinum longifolium* L. [NEHU-10861]	Tiew Lili (Kh), Gorur mod (B)	H	PR	BL	Fever, bone fractured	T, O	Infusion, paste	250 ml mixed with 1 *l* of tea, given twice a day for fever
**Anacardiaceae**								
*Mangifera indica* L. [NEHU-10869]	Soh-pieng (Kh), Aam (B)	T	PR	FR	Constipation, indigestion	O	Paste	1 kg ripe fruit mixed with *Psyllium* husk directly per day
*Rhus javanica* L. [NEHU-10870]	Soh-ma (Kh)	T	PR	FR	Foot and mouth disease	O	Direct feeding	NA
**Apiaceae**								
*Coriandrum sativum* L. [NEHU-10855]	Dhunia (Kh), Dhoina (B)	H	AN	LF, SE	Ectoparasite control, diarrhoea	T, O	Paste, direct feeding	3-5 bundles of leaves and 10 *g* of soaked seeds mixed with bark of *Terminalia arjuna* fodder, given twice a day
*Centella asiatica* (L.) Urb. [NEHU-10856]	Khliangsyiar (Kh), Mani muni pata (B)	H	AN	LF	Skin infection, neurological disorder, dysuria	T, O	Infusion	NA
*Hydrocotyle rotundifolia* Roxb. [NEHU-10857]	Mani muni pata (B)	H	AN	LF	Health tonic	O	Direct feeding	NA
*Trachyspermum ammi* (L.) Sprague [NEHU-10858]	Ajjwain (B)	H	AN	SE	Indigestion, bloat, after parturition	O	Paste	20 *g* mixed with jiggery for daily basis
**Asparagaceae**								
*Asparagus racemosus* Willd. [NEHU-10872]	Shatavari (B)	H	PR	RT	Increase lactation	O	Decoction	100 ml mixed with fodder in daily basis
*Chlorophytum arundinaceum* Baker [NEHU-10873]	–	H	PR	WP	Ectoparasite control, health tonic	T, O	Decoction	NA
**Asteraceae**								
*Artemisia vulgaris* L. [NEHU-10852]	Dieng tlio (Kh)	S	PR	LF, FL	Skin infection	T	Paste	100 *g* is mixed 20 ml of *Eucalyptus* oil applied directly twice a day for two weeks
*Taraxacum officinale* Wigg. [NEHU-10853]	–	H	AN	RT	Fever	T	Paste	NA
*Tagetes erecta* L. [NEHU-10854]	Tiew mangor (Kh)	H	AN	LF	Wound treatment	T	Paste	100 *g* of dried leaves mixed with *50* g of turmeric powder given once a day
**Boraginaceae**								
*Cynoglossum zeylanicum* (Vahl) Brand [NEHU-10877]	–	H	AN	WP	Wound treatment, conjunctivitis	T	Decoction	NA
**Brassicaceae**								
*Brassica campestris* L. [NEHU-10878]	Sla-tyrso (Kh)	H	BA	SE	Skin infection, cough and cold, conjunctivitis	T	Paste	250 *g* of seed is mixed with 50 *g of* sulphur, *Piper longum* L. and diluted with water for daily basis
**Cannabinaceae**								
*Cannabis sativa* L. [NEHU-10883]	Bhaang gaach (B)	H	AN	LF	Dysentery	O	Juice	200 ml mixed with fodder, given twice a day
**Caricaceae**								
*Carica papaya* L. [NEHU-10886]	Pap pay (B)	T	PR	SE	Health tonic	O	Direct feeding	250 *g* mixed with jiggery, given once a day
**Caryophyllaceae**								
*Drymaria cordata* (L.) Willd. ex Schult [NEHU-10885]	–	H	AN	LF	Indigestion	O	Direct feeding	NA
**Commeliaceae**								
*Cyanotis tuberosa* (Roxb.) Schult. & Schult. f. [NEHU-10880]	–	H	AN	ST	Fever, cough and cold	O	Decoction	500 ml added common salt, given thrice a day
**Convolvulaceae**								
*Cuscuta reflexa* Roxb. [NEHU-10881]	Jawieh (Kh), Sonalu lata (B)	P	PR	WP	Bone fracture, foot and mouth disease, indigestion	T, O	Paste	NA
**Crassulaceae**								
*Bryophyllum pinnatum* (Lam.) Oken [NEHU-10879]	Pathori pata (B)	H	PR	LF	Wound treatment, dysuria	O	Infusion	500 ml added black salt, given twice a day
**Cyperaceae**								
*Cyperus rotundus* L. [NEHU-10884]	Mutha gaach (B)	H	PR	LF	Food poisoning	O	Infusion	500 ml added with 5 *l* of lukewarm water, given twice a day
**Equisetaceae**								
*Equisetum diffusum* D. Don [NEHU-10887]	Haddi bhanga (B), Japung blei (Kh)	H	PR	LF	Bone fracture	T	Paste	NA
**Ericaceae**								
*Rhododendron arboreum* Sm. [NEHU-10892]	Tiew Thuin (Kh)	T	PR	FL	Diarrhoea and dysentery	O	Infusion	500 ml added with 5 *l* water, given thrice a day
*Lyonia ovalifolia* (Wall.) Drude [NEHU-10882]	Diengla-samiang (Kh)	T	PR	RT	Wound treatment	T	Decoction	NA
**Euphorbiaceae**								
*Phyllanthus urinaria* L. [NEHU-10888]	–	H	PR	LF	Dysuria	O	Infusion	500 ml added with water given twice a day
*Phyllanthus emblica* L. [NEHU-10889]	Soh maleng (Kh), Amolokhi (B)	T	PR	FL, LF	Fever, cough, anthrax	O	Powder	50 *g* (mixed with *Ocimum basilicum* L. leaves and honey) given twice a day
*Ricinus communis* L. [NEHU-10890]	Sla ryndia (Kh), Bharrond (B)	S	PR	LF, SE	Skin infection, constipation	T, O	Paste and powder	20 *g* seed powder directly with fodder once a day
*Euphorbia hirta* L. [NEHU-10891]	Dudhejhar (B)	H	AN	WP	Increase lactation	O	Direct feeding	Ca. 10 plant given directly with banana leaf
**Fabaceae**								
*Albizia lebbeck* Benth. [NEHU-10893]	Koroi gaach (B)	T	PR	BR	Fever, ectoparasite control	T, O	Decoction	NA
*Bauhinia variegata* L. [NEHU-10894]	Dieng Long (Kh), kancchan mala (B)	T	PR	LF, FL	Conjunctivitis, diarrhoea, dysentery	O	Infusion	200 ml mixed with the fodder, given twice a day
*Butea monosperma* (Lam.) Kuntze [NEHU-10895]	Polash gaach (B)	T	PR	FL, BR	Bloat, deworming, dysuria	O	Decoction	1 *l* added with the jiggery, given once a day
*Cassia fistula* L. [NEHU-10896]	Sonuru gaach (B)	T	PR	SE, FL	Respiratory disease, anthrax	O	Powder	200 *g* dried pod/fruit pulp is mixed with fodder, given twice a day
*Erythrina suberosa* Roxb. [NEHU-10897]	Mandal gaach (B)	T	PR	LF	Foot infection	T	Paste	NA
*Entada scandens* Benth. [NEHU-10898]	Mei-nok (Kh), Ghila gaach (B)	C	PR	SE	Fever	T	Powder	100 *g* mixed with tea for thrice a day
*Mimosa pudica* L. [NEHU-10899]	Lajja potti (B)	H	PR	LF	Bone fracture	T	Paste	NA
**Lamiaceae**								
*Gmelina arborea* Roxb. [NEHU-10900]	Ghambhari gaach (B)	T	PR	BR	Fever	T	Decoction	50 ml mixed with fodder for twice a day
*Leucas zeylanica* Br. [NEHU-10901]	Chondro kolosh (B)	H	AN	LF	Fever, cold and cough	O	Infusion	200 ml mixed with honey and fodder for once a day
*Mentha piperata* L. [NEHU-10903]	Pudina (Kh), Pudinhara (B)	H	PR	LF	Indigestion, fever	O	Infusion	1 *l* is mixed with water for twice a day
*Ocimum basilicum* L. [NEHU-10904]	Tulsi (Kh)	H	AN	LF	Snake bite	T	Paste	NA
*Ocimum gratissimum* L. [NEHU-10905]	Ram tulsi (B)	H	AN	LF	Indigestion, ectoparasite control	T, O	Juice	NA
*Ocimum tenuiflorium* L. [NEHU-10906]	Krishna tulsi (B)	H	AN	SE	Constipation	O	Powder	100 *g* (added with sugar and salt) mixed with fodder for twice a day
**Malvaceae**								
*Sida acuta* Burm. f. [NEHU-10914]	Jhaaru gaach (B)	H	PR	WP	Diarrhoea	O	Direct feeding	NA
**Melastomataceae**								
*Osbeckia nepalensis* Hook. f. [NEHU-10915]	Ja lyngkthem (Kh)	S	PR	LF, FR	Food poisoning	O	Decoction	500 ml is mixed with water for twice a day
**Meliaceae**								
*Azadirachta indica* A juss. [NEHU-10907]	Sla nim (Kh), Neem gaach (B)	T	PR	WP	Skin infection, ectoparasite control, food poisoning	T, O	Decoction	500 ml is mixed with 20 *l* water, used externally and 50 ml given directly once a day for food poisoning
*Melia azedarach* L. [NEHU-10908]	Dieng-jah-rasang (Kh), Maha nim (B)	T	PR	LF	Skin infection, foot and mouth disease	T, O	Paste	NA
**Moraceae**								
*Artocarpus lakoocha* Roxb. [NEHU-10909]	Dieng-soh-ram (Kh), Champ kathool (B)	T	PR	LF	Mouth infection	O	Direct feeding	NA
*Artocarpus heterophyllus* Lam. [NEHU-10910]	Dieng-soh-phan (Kh), Kathool (B)	T	PR	LF	Increase lactation	O	Direct feeding	NA
*Ficus religiosa* L. [NEHU-10911]	Peepal gaach (B)	T	PR	ST	Constipation, respiratory diseases	O	Decoction	1 *l* (added *Plantigo* seed power) with lukewarm water once a day
*Ficus bengalensis* L. [NEHU-10912]	Bood gaach (B)	T	PR	RT	Diarrhoea, dysentery, skin infection	O	Decoction/Paste	NA
*Ficus hispida* L. f. [NEHU-10913]	Jagooth dombol (B)	T	PR	LF	Indigestion	O	Direct feeding	NA
**Musaceae**								
*Musa paradisiaca* L. [NEHU-10916]	Kola gaach (B)	H	PR	LF	Mastitis	T	Paste	NA
**Oxalidaceae**								
*Oxalis corniculata* L. [NEHU-10917]	Soh-khia-khnai (Kh), Ambuli pata (B)	H	AN	LF	Health tonic, dysentery	O	Direct feeding	1 kg/day feeding directly
**Papavareaceae**								
*Argemone maxicana* L. [NEHU-10928]	–	H	AN	LF	Joint pain	T	Paste	NA
**Pinaceae**								
*Pinus kesiya* Royle ex. Gordon [NEHU-10927]	Sal gaach (B), Dieng Kseh (Kh)	T	PR	BR	Health tonic	T	Oil	50 ml mixed with coconut oil, used daily basis
**Plantaginaceae**								
*Plantago major* L. [NEHU-10926]	Skhor blong (Kh)	H	PR	LF	Skin infection	T	Infusion	NA
**Poaceae**								
*Cynadon dactylon* (L.) Pers. [NEHU-10918]	Durbha pata (B)	H	PR	LF	Mastitis, conjunctivitis	T	Infusion	NA
*Zea mays* L. [NEHU-10919]	Riewhadem (Kh), makooi (B)	H	AN	SE	Increase lactation	O	Powder	1 kg mixed with fodder, given once in every day
*Saccharum officinarum* L. [NEHU-10920]	Pai (Kh), Gandhari (B)	H	PR	WP	Increase lactation	O	Direct feeding	NA
*Oryza sativa* L. [NEHU-10921]	Kba (Kh), Dhan (B)	H	AN	SE	Diarrhoea	O	Direct feeding	500 *g* given directly once in every day
*Cymbopogon nardus* (L.) Rendle [NEHU-10922]	–	H	PR	LF	Health tonic	O	Direct feeding	NA
*Thysanolaena maxima* (Roxb. ex Hornem.) Honda [NEHU-10923]	Synsar (Kh)	H	PR	LF	After parturition	T	Paste	NA
**Polygonaceae**								
*Rumex nepalensis* Spreng [NEHU-10924]	Jungali palong (B)	H	PR	RT	Food poisoning, fever	O	Decoction	1 *l* (added with salt and sugar) given twice a day
*Polygonum strigosum* R. Br. [NEHU-10925]	Mutha (B)	H	AN	WP	Health tonic	O	Direct feeding	NA
**Rhamnaceae**								
*Ziziphus jujube* Mill. [NEHU-10939]	Soh-broi (Kh)	T	PR	LF	Skin infection	T	Paste	NA
**Rosaceae**								
*Prunus persica* (L.) Batsch [NEHU-10929]	Soh-pharang (Kh), Aru fal (B)	T	PR	LF	Conjunctivitis	T	Infusion	NA
*Rosa indica* L. [NEHU-10931]	Tiew kulab (Kh), Gulab ful (B)	S	PR	FL	Increase lactation	O	Direct feeding	100 *g* of dried flowers mixed with fodder, given daily basis
*Rubus ellipticus* Smith. [NEHU-10932]	Sia-soh-pru (Kh)	S	PR	ST	Bone fracture	T	Young stem paste	NA
**Rubiaceae**								
*Paederia foetida* L. [NEHU-10937]	Gondho lota (B)	C	PR	LF	Dysentery, indigestion	O	Juice	250 ml is added with rice and wheat husks, once a day for 7 days
*Rubia cordifolia* L. [NEHU-10938]	Soh-misem (Kh), manjistha (B)	C	PR	RT, LF	Food poisoning, skin infection	T, O	Decoction and leaf juice	NA
**Rutaceae**								
*Murraya koenigii* Spreng [NEHU-10933]	Curry pata (B)	T	PR	LF	Indigestion	O	Juice	100 ml mixed with fodder, given twice a day
*Aegle marmelos* Correa [NEHU-10934]	Soh-bel (Kh), Bel (B)	T	PR	FR	Indigestion, bloat, fever	O	Direct feeding	Few ripe fruits on daily basis
*Citrus limon* (L.) Burm.f. [NEHU-10935]	Soh-jaw (Kh), Nibu (B)	T	PR	FR	Indigestion	O	Direct feeding	2-5 ripe fruits on daily basis
*Zanthoxylum armatum* Roxb. [NEHU-10936]	Jai-ur-khlaw (Kh), Gul morich (B)	T	PR	BR, LF	Indigestion, endoparasite control	T	Bark pastes and leaf Infusion	NA
**Solanaceae**								
*Capsicum annuum* L. [NEHU-10940]	Soh-mynken (Kh), Kacha lonka (B)	H	AN	FR	Haemorrhagic septicaemia	O	Decoction	500 ml added with salt is given directly once a day for 3 days
*Solanum esculentum* Mill. [NEHU-10941]	Soh-saw (Kh), Tomato (B)	H	AN	FR	Conjunctivitis	T	Juice	NA
*Solanum nigrum* L. [NEHU-10942]	Kynbat iong (Kh), Tita begun (B)	H	AN	LF	Food poisoning, dysuria	O	Direct feeding	NA
*Datura metel* L. [NEHU-10943]	Syntiew-Khlieh-biej (Kh), Dhutra gaach (B)	S	AN	LF, SE	Skin infection, insect bite	T	Paste	NA
**Theaceae**								
*Camellia sinensis* (L.) Kunteze [NEHU-10945]	Sla-Scha (Kh), Cha-patti (B)	S	PR	LF	Fever	O	Infusion	1 *l* is mixed with ginger and jiggery given once a day
*Schima wallichi* Choisy [NEHU-10946]	Dieng-ngan (Kh), Maakri sal gaach (B)	T	PR	BR	Wound treatment, endoparasite control	T, O	Powder	100 *g* mixed with wheat husks, given twice a day at morning time
**Thelypteridaceae**								
*Christella parasitica* H. L. [NEHU-10944]	–	F	PR	LF, RH	Food poisoning	O	Decoction	200 ml mixed with turmeric and fodder, given twice a day
**Verbenaceae**								
*Lippia alba* (Mill.) N.E.Br. ex Britton & P. Wilson [NEHU-10947]	Soh-pung-khleh (Kh)	S	PR	FL, LF	Fever, skin infection	T, O	Paste	NA
*Clerodendron serratum* L. [NEHU-10948]	Rilong-phlang (Kh)	S	PR	LF, FL	Food poisoning	O	Juice	1 *l* mixed with water, given twice a day
**Xanthorrhoeaceae**								
*Aloe barbediensis* L. [NEHU-10949]	Kynbat-khluid-ding (Kh), Gritha kumara (B)	H	PR	ST	Endoparasite control	O	Decoction	500 ml added with fodder, given twice a day
**Zingiberaceae**								
*Curcuma caesia* Roxb. [NEHU-10950]	Kala haldi (B)	H	PR	RH	Dysentery	O	Paste	250 ml added with jiggery given thrice a day
*Curcuma longa* L. [NEHU-10951]	Shymai (Kh), Hauldi (B)	H	PR	RH	Mastitis, wound treatment, joint pain	T	Paste	NA
*Zingiber officinale* Roscoe [NEHU-10952]	Ada (B)	H	PR	RH	Indigestion	O	Decoction	250 ml mixed with 1 *l* of water and is given directly after having fodder

***Legends:*** H = Herb, S = Shrub, T = Tree, P = Parasite, C = Climber, F = Fern, AN = Annual, BA = Biannual, PR = Perennial, LF = Leaf, RT = Root, SE = Seed, ST = Stem, BL = Bulb, BR = Bark, ST = Stem, DP = Direct feeding, FR = Fruit, FL = Flower, RH = Rhizome, WP = Whole plant, O = Oral, T = Topical, NA = Not available.

### 3.3 Informant consensus factor

Informant consensus factor (Fic) values were determined to know the uniformity among the information provided by different informants. The Fic values ranged from 0.50 to 1.00 with an average value of 0.74 (Fig. 2). The highest Fic value of 1.00 with 4 use-reports for 1 species were obtained for neurological disorder followed by foot & mouth diseases (Fic = 0.91; 5 species and 47 use-reports) and deworming (Fic = 0.90; 4 species and 30 use-reports). *Centella asiatica* (L.) Urb., *Melia azedarach* L. and *Zanthoxylum armatum* Roxb. were the species that accounted for the highest consensus for neurological disorder, foot & mouth diseases and deworming. The lowest agreement between the informants was observed in respiratory diseases (Fic = 0.50; 4 species and 7 use-reports) followed by fever (Fic = 0.52; 14 species and 28 use-reports) and skin infection (Fic = 0.60; 18 species and 44 use-reports). Skin infection had the lowest Fic (0.60) after respiratory and fever but this ailment category ranked first in the number of taxa (18) and fifth in the number of use reports (44) attributed to this category.

### 3.4 Fidelity level

Plants frequently used for curing the major categories of ailment as claimed by different informants were analysed by using fidelity level (FL %) based on use reports. The fidelity level (FL %) values of species that were declared to be used by informants against the corresponding ailment are depicted in Table 4. The values ranged from 42.85% to 100% with an average value of 71.95%. After the data analysis, the FL value with 100% was found in *Aegle marmelos* Correa, followed by *Tagetes erecta* L. (94.11%), *Melia azedarach* L. (87.50%) and *Camellia sinensis* (L.) Kunteze (85.71%). The lowest FL value was recorded in *Cassia fistula* L. (42.85%) followed by *Centella asiatica* (L.) Urb. (50.00%) and *Bauhinia variegata* L. (54.54%). The highest FL value (100%) was obtained from indigestion, wound treatment (94.11%) and foot and mouth disease (87.50%) while the lowest FL value was recorded for anthrax (42.85%), neurological disorder (50.00%) and conjunctivitis (54.54%).

**Table 3:** List of species with specific bioactive compounds, isolated from various parts.

Scientific name	Bioactive compounds	Source
*Achyranthus aspera* (Lam.) Griseb.	Triterpenoid saponins (oleonolic acid), ecdysterone, achyranthine, betaine, pentatriaontane, hexatriacontane, tritriacontane	[32]
*Acorus calamus* L.	Alpha (α)-asarone, beta (β)-asarone, acoradin, eugenol, β-sitosterol, azulene	[33]
*Aegle marmelos* Correa	Marmelosin, aegeline, lupeol, marmin, fagarine, tannin	[34]
*Albizia lebbeck* Benth.	Albiziasaponins, lupenone, freidelin, lupeol, β sitosterol- 3-O-glucoside, stigmasterol -3-O-glucoside, luteolin, rutin	[35]
*Allium cepa* L.	Organic sulphur, flavonoids, polyphenols	[36]
*Allium sativum* L.	Allicin, alliin, diallyl sulfides, ajoene, S-allyl-cysteine	[37]
*Aloe vera* (L.) Burm.f.	Anthraquinone, tannin, saponin, flavonoids, terpenoids, octadecanoic acid, tricosane, 1-octadecanol	[38]
*Alternanthera philoxeroides* (Mart.) Griseb.	Glycosylated flavonoids, saponins, betalains	[39]
*Amaranthus viridis* L.	Erucic acid, 3-Hydroxy-N-methylphenethylamine, n-Hexadecanoic acid, Cystamine	[40]
*Andrographis paniculata* (Burm.f.) Wall. ex Nees	Diterpenoids (andrographolide), flavones, polyphenols	[41]
*Argemone maxicana* L.	Alkaloids (berberine, isocorydine, protopine, dehydrocorydalmine, jatrorrhizine, columbamine) terpenoids, flavonoids, phenolics, long-chain aliphatic compounds	[42]
*Artemisia vulgaris* L.	β-caryophyllene, α-zingiberene, borneol, α-curcumin	[43]
*Artocarpus heterophyllus* Lam.	Phenolics, flavonoids, terpenoids, stilbenoids, carbohydrates, protein, amino acid, vitamin, minerals	[44]
*Artocarpus lakoocha* Roxb.	β- sitosterol, diethyl phthalate, 3,4-dihydroxymandelic acid, 9-octyl eicosane, lupeol	[45]
*Asparagus racemosus* Willd.	Asparagamine A, racemosol, hydroxycinnamic acids, saponins	[46]
*Azadirachta indica* A juss.	Azadirachtin, nimbin, sodium nimbinate, salannin, quercetin	[47]
*Bauhinia variegata* L.	Lupeol, β-sitosterol, kaempferol, quercetin	[48]
*Brassica campestris* L.	Glucosinolates, phenolics, ascorbic acid, carotenoids	[49]
*Bryophyllum pinnatum* (Lam.) Oken	Bryophylluside, kaempfrol, rutin, quercetin 3-O-α-l-arabinopyranosyl (1 → 2) α-l-rhamnopyranoside, quercitrin, β-sitosterol	[50]
*Butea monosperma* (Lam.) Kuntze	Cajanin, Butin, flavonoids, medicarpin	[51]
*Camellia sinensis* (L.) Kunteze	Flavonoids, phenolic compounds, alkaloids, tannins, volatile constituents, amino acids	[52]
*Cannabis sativa* L.	Delta-9-tetrahydrocannabinol (THC), nonpsychoactive cannabidiol (CBD)	[53]
*Capsicum annum* L.	Carotenoids, tocopherols, capsaicinoids (capsacicin)	[54]
*Carica papaya* L.	Phenolics, vitamin E, carotenoids	[55]
*Cassia fistula* L.	Phenolic antioxidants (anthraquinones, flavonoids, flavan-3-ol derivatives)	[56]
*Centella asiatica* (L.) Urb.	Asiatic acid, asiaticoside, madecassic acid, madecassoside	[57]
*Chenopodium album* L.	Polyphenol, alkaloids, glycosides, saponins, flavonoids	[58]
*Chlorophytum arundinaceum* Baker	Fatty acids, common sterol stigmasterol, one furostanol saponins	[59]
*Christella parasitica* H. L.	Terpenoids, alkaloids, tannins, saponins, flavonoids	[60]
*Citrus limon* (L.) Burm.f.	Phenolic compounds as well as vitamins, minerals, dietary fibre, essential oils, carotenoids	[61]
*Clerodendron serratum* L.	D-mannitol, cleroflavone, apigenin, verbascoside, oleanolic acid, clerodermic acid, γ-sitosterol, β-sitosterol	[62]
*Coriandrum sativum* L.	Linalool, anethole, decanal, geraniol, geranyl acetate, tetradecenol, undecanol, p-Cymene, α-Phellandrene	[63]
*Crinum longifolium* L.	Crinine-type alkaloids	[64]
*Curcuma caesia* Roxb.	germacrone, zerumbone, furanodienone, curzerenone, curcumenol, zederone, dehydrocurdione, curcuzederone, 4-diepoxide, aerugidiol, zedoarondiol	[65]
*Curcuma longa* L.	Curcumin, α-turmerone, germacrone, ar-curcumene, β–turmerone, α-zingiberene	[66]
*Cuscuta reflexa* Roxb.	Phenolics (kaempferol, quercetin), coumarins, flavonoid glycosides	[67]
*Cyanotis tuberosa* (Roxb.) Schult. & Schult. f.	Anthraquinones, saponins, tannins, glycosides, terpenoids	[68]
*Cymbopogon nardus* (L.) Rendle	Elemol, citronellol, naphthalene	[69]
*Cynadon dactylon* (L.) Pers.	Glycerin, thymol, conhydrin, linoleic acid, octadecadienoyl acid, diazoprogesterone	[70]
*Cynoglossum zeylanicum* (Vahl) Brand	Alkaloids, catechin, coumarin, tannin, phenols, saponins, steroid, flavonoid, glycoside, xanthoprotein	[71]
*Cyperus rotundus* L.	Alkaloids, phenols, flavonoids, terpenes	[72]
*Datura metal* L.	β-sitosterol, atropine, hyoscyamine, fastusic acid, triterpene, daturanolone, daturadiol	[73]
*Drymaria cordata* (L.) Willd. Ex Schult	Glycolipids (stigmasterol, stigmasteryl glucoside, cerebroside, glucocerebroside)	[74]
*Entada scandens* Benth.	Phenolics, phytic acid	[75]
*Equisetum diffusum* D. Don	Silica acid, caffeic acid, ferulic acid, dimethylsulphone (C_2_H_6_0_2_S), alkaloid palustrine (C_17_H_29_H_3_0_2_)	[76]
*Erythrina suberosa* Roxb.	α-hydroxyerysotrine, 4′-methoxy licoflavanone (MLF), alpinumisoflavone (AIF), wighteone	[77]
*Euphorbia hirta* L.	Alkanes, triterpenes, phytosterols, tannins, polyphenols, flavonoids	[78]
*Ficus bengalensis* L.	Bengalenosides, coumarin, sterols, tiglic acid esters, alpha-d-glucose and meso-inositol	[79]
*Ficus hispida* L. f.	Sesquiterpenoids and triterpenoids, flavonoids, coumarins, phenylpropionic acids, alkaloids, steroids	[80]
*Ficus religiosa* L.	Phytosterols, amino acids, furanocoumarins, phenolics, hydrocarbons, aliphatic alcohols, volatile components	[81]
*Gmelina arborea* Roxb.	Arboreal, tyrosol, iridoids, phenylpropanoid glycoside, premnazole, martynoside, gmelinol, umbelliferone	[82]
*Hydrocotyle rotundifolia* Roxb.	Hydrocotyloside I-VII, hydrocosisaponin, quercetin, camphene, catechin, epicatechin, ferulic acid, α-humulene	[83]
*Justicia adhatoda* L.	Quinazoline alkaloid, vasicine (1, 2, 3, 9-tetrahydropyrrole [2,1-b] quinozolin-3-ol, C_11_H_12_N_2O_), vasicinone	[84]
*Leucas zeylanica* Br.	Catechol, ethyl caffeate, isoeugenin, darendoside B, isolariciresinol, curcasinlignan B, formononetin	[85]
*Lippia alba* (Mill.) N.E.Br. ex Britton & P. Wilson	Carvone, limonene, bicyclosesquiphellandrene, piperitenone, piperitone and β-bourbonene	[86]
*Lyonia ovalifolia* (Wall.) Drude	Phenolics, flavonoids, glycosides, tannins, xanthoprotein, quinones, saponins	[87]
*Mangifera indica* L	Polyphenols, anthocyanins, carotenoids, derivatives of gallotannins	[88]
*Melia azedarach* L.	Flavonoid glycosides, limonoids, β-Sitosterol, quercetin, meliacine	[89]
*Mentha piperata* L.	Flavonoids, volatile oils (methanol and methone), lutolin, hesperidins	[90]
*Mimosa pudica* L.	Mimosine, tannins, steroids, flavonoids, triterpenes, glycosylflavones	[91]
*Murraya koenigii* Spreng	Lutein, tocopheral, carotene, koenimbine, isomahanimbine	[92]
*Musa paradisiaca* L.	Phenolics, β-carotene, tannins, ascorbic acid, saponins, lycopene, phytosterols	[93]
*Ocimum basilicum* L.	Luteolin, eriodictyol, eugenol, methyl cinnamate, rosmarinic acid, caryophyllene	[94]
*Ocimum gratissimum* L.	Oleanolic acid, ellagic acid, epicatechin, rosmarinic acid, apigenin, gallic acid, quercetin, rutin, kaempferol, essential oils (camphene, β-caryophyllene, sabinene, β-myrcene, limonene)	[95]
*Ocimum tenuiflorium* L.	3,4-dimethoxycinnamic acid, caffeic acid, diosmetin, luteolin, kaempferol, rosmarinic acid, apigenin, genistein	[96]
*Oryza sativa* L.	Phenolic acids, flavonoids, anthocyanins, proanthocyanidins, tocopherols, tocotrienols, γ-oryzanol and phytic acid	[97]
*Osbeckia nepalensis* Hook. f.	Tannin,β-sitosterol, quercetin, rutin, flavonoid, minerals	[98]
*Oxalis corniculata* L.	Flavonoids, alkaloids, tannins, phenols	[99]
*Paederia foetida* L.	Asperuloside, paederosidic acid, phenols, alkaloid, volatile oil, sitosterols, ellagic acid, lignans, iridoids, triterpenoids, urosilacid, epifriedelinol	[100]
*Peristrophe bivalvis* Merr.	Pelargonidin, zizilan	[101]
*Phyllanthus emblica* L.	Tannins, flavonoids, saponins, terpenoids, alkaloids, ascorbic acid	[102]
*Phyllanthus urinaria* L.	Lignans, tannins, flavonoids, phenolics, terpenoids	[103]
*Pinus kesiya* Royle ex. Gordon	Catechin, caffeic acid, ferulic acid, taxifolin	[104]
*Plantago major* L.	Iridoid glucosides, phenylethanoid glycosides, 10-acetylarborescoside, plantainoside G	[105]
*Polygonum strigosum* R. Br.	Anthraquinones, flavonoid, quercetin, kaempferol, luteolin	[106]
*Prunus persica* (L.) Batsch	Polyphenols, flavonoids, anthocyanins	[107]
*Rhododendron arboreum* Sm.	Quercetin-3-rhamnoside, phenols, rutin, coumaric acid	[108]
*Rhus javanica* L.	Gallotannin, flavonoids, urushiols, terpenoids	[109]
*Ricinus communis* L.	Ribitol, 3-ethoxy-1, 2-propanediol, p-dioxane-2, dodecanoic acid, cetene, geranyl isovalerate, phenol, picrotoxinin	[110]
*Rosa indica* L.	Quinic acid, pyrogallol, 5-hydroxymethylfurfural	[111]
*Rubia cordifolia* L.	Anthraquinones, glycosides, flavonoids, steroids, phenols, saponins	[112]
*Rubus ellipticus* Smith.	Gallic acid, rutin, phenolics, flavonoids	[113]
*Rumex nepalensis* Spreng	Anthraquinone, naphthalene, rumexneposides, torachrysone, glucopyranoside, citreorosein, nepodin, rhapontigenin	[114]
*Saccharum officinarum* L.	Flavonoids (apigenin, luteolin and tricin derivatives) and phenolics (hydroxycinnamic, caffeic and sinapic acid)	[115]
*Schima wallichi* Choisy	Polyphenolic, quinones, anthocyanosides, coumarin derivatives, kaempferol-3-O-rhamnoside	[116]
*Sida acuta* Burm. f.	Cryptolepine, kaempferol glycosides, anthocyanins	[117]
*Solanum esculentum* Mill.	Lycopene, β-carotene, ascorbic acid, polyphenols	[118]
*Solanum nigrum* L.	Alkaloids, flavonoids, glycosides, phytosterols, phenolic compounds, tannins, saponins	[119]
*Tagetes erecta* L.	Flavanols (especially quercetagetin glycosides), polyphenols (hydroxybenzoic and hydroxycinnamic acid derivatives), β-caryophyllene	[120]
*Taraxacum officinale* Wigg.	Sesquiterpene lactones (mainly taraxinic acid glucoside, TA-G), triterpene acetates, phenolic inositol esters	[121]
*Thysanolaena maxima* (roxb. Ex Hornem.) Honda	Sitostenone, 4-hydroxy cinnamic acid, 4-hydroxy benzaldehyde, stigmasterol	[122]
*Trachyspermum ammi* (L) Sprague	Carbohydrates, glycosides, saponins, phenolic compounds	[123]
*Zanthoxylum armatum* Roxb.	Linalool, β-ocimene, cinnamic acid, limonene, α-terpinolene, germacrene	[124]
*Zea mays* L.	Flavonoids, tannins, phlobatannins, phenols, alkaloids, cardiac glycosides	[125]
*Zingiber officinale* Roscoe	gingerols, flavonoids, shogaols, diarylheptanoids, diterpenoids, phenylbutenoids, sesquiterpenoids	[126]
*Ziziphus jujube* Mill.	3-O-(*trans*-p-coumaroyl)-alphitolic acid, 3β-O-(*trans*-p-coumaroyl)-maslinic acid, pomonic acid, benthamic acid, oleanic acid, quercetin, traumatic acid, magnoflorine	[127]

**Table 4:** Fidelity level (Fl %) of most frequently used plants for different ailment categories (Total informants = 52).

Scientific name	Ailment Category	SF	TF	FL (%)
*Aegle marmelos* Correa	Indigestion	9	9	100.00
*Tagetes erecta* L.	Wound treatment	32	34	94.11
*Melia azedarach* L.	Foot and mouth disease	14	16	87.50
*Camellia sinensis* (L.) Kunteze	Fever	12	14	85.71
*Euphorbia hirta* L.	Increase lactation	25	30	83.33
*Azadirachta indica* A juss.	Food poisoning	14	17	82.35
*Cynadon dactylon* (L.) Pers*.*	Mastitis	8	10	80.00
*Phyllanthus emblica* L.	Cold and cough	27	36	75.00
*Solanum nigrum* L.	Dysuria	9	12	75.00
*Rhododendron arboreum* Sm.	Diarrhoea and dysentery	23	31	74.19
*Azadirachta indica* A juss.	Skin infection	21	29	72.41
*Trachyspermum ammi* (L.) Sprague	Constipation and bloat	16	23	69.56
*Capsicum annuum* L.	Haemorrhagic septicaemia	2	3	66.66
*Ocimum basilicum* L.	Poisonous bites	10	15	66.66
*Amaranthus viridis* L.	Health tonic	20	31	64.51
*Zanthoxylum armatum* Roxb.	Deworming	17	27	62.96
*Cuscuta reflexa* Roxb.	Bone fracture and joint pain	8	13	61.53
*Cassia fistula* L.	Respiratory diseases	4	7	57.14
*Bauhinia variegata* L.	Conjunctivitis	6	11	54.54
*Centella asiatica* (L.) Urb.	Neurological disorder	2	4	50.00
*Cassia fistula* L.	Anthrax	3	7	42.85

***N.B****:* FL = Fidelity Level, SF = is the number of informants who independently cited the importance of a species for treating a particular disease, and TF = total number of citations.

### 3.5 Documentation of bioactive compounds

The collected ethnoveterinary plants possessed a number of pharmacological properties and produces a wide range of bioactive chemical compounds through their secondary metabolism. Available published literature (viz., Google Scholar (www.scholar.google.co.in), Web of Science (http://thomson reuters.com), PubMed (www.ncbi.nlm.nih.gov/pubmed), CAB direct (www.cabdirect.org), SciFinder (www.cas.org/products/scifinder) and Dictionary of Natural Products (www.dnp. chemnetbase.com) revealed that all the taxa used for the treatment of various cattle diseases contain a wide range of valuable chemical compounds (Table 3). These compounds are reported to have beneficial effects on the prevention of various diseases, depending on which species are used and how much quantity is taken.

## 4 Discussion

Meghalaya a part of the Indo Burma biological hotspot, sustains a diverse array of traditional knowledge which spreads across food, medicine, fuel, architecture and agro-forestry. Inhabitants of Meghalaya have a close association with nature as they are used plants for medicine in curing numerous ailments, due to its dominance of ethnic people, who are reliant on forest products since time eternal [128,129]. The region has vast pharmaceutical and commercial potentials as the knowledge of plants were inbuilt in their deep-rooted culture, which is being transferred from generation to generation. Hence, an attempt has been made in the present study to document the ethnoveterinary medicinal knowledge, focusing primarily to find out the gap and scope in further ethnobotanical research in the region for searching novel bioactive compounds. The present study reveals that plants and their byproducts are the most regular and easily available medicine used by traditional herbal healers and animal keepers on various ailments of their livestock [[130], [131], [132]]. The current findings are aligned with the various studies conducted in other parts of India on various ethnic groups [14,[19], [20], [21], [22],^24^,^26^]. Plants like *Zingiber officinal*, *Azadiracca indica*, *Allium sativum*, *Acorus calamus*, *Curcuma longa*, *Andrographis paniculata*, etc., used by Khasi people for the treatment of the livestock were similarly found to be used by other ethnic people of the country [[133], [134], [135], [136], [137], [138]], which signifies the recognition of these species as medicinal importance for noble drug findings [134,139]. The paste and powder are the most common mode of application of herbal medicines taken either orally or externally as seen in the present study have a similar finding cited in the literature as well [14,138,140,141]. Diseases recorded like foot and mouth disease, diarrhoea, bloating, etc. in the present study are also frequent among cattle in various states of India [23,[142], [143], [144], [145]]. In the present study, the species *Centella asiatica*, *Melia azedarach* and *Zanthoxylum armatum* were commonly used for the treatment of neurological disorders, foot & mouth diseases and deworming, resulting in the highest Fic value [146]. The plants with the highest Fic are because of their systematic selection and information procedures provided by informants [131]. The species with the highest FL of 100% were *Aegle marmelos*, *Tagetes erecta*, *Melia azedarach*, *Camellia sinensis* and for indigestion, wound treatment, foot and mouth diseases and fever [131,147,148]. So, we recommend these species for further phytochemical and pharmacological studies for the development of new, cheap, effective, and eco-friendly herbal formulations for healthcare management [[149], [150], [151]]. Priority should be given to the plants with the highest consensus factor and fidelity level, which could potentially guide the search for new pharmaceutical and commercial products of universal interest.

### 4.1 Scientific validation of medicinal plants

The medicinal plants with the highest consensus factor and fidelity level were compared with previously reported studies for biological activities and bioactive constituents responsible for their therapeutic properties. These species are *Aegle marmelos* Correa, *Tagetes erecta* L., *Melia azedarach* L., *Centella asiatica* (L.) Urb., *Zanthoxylum armatum* Roxb. and *Ocimum basilicum* L.

**Fig. 7:**
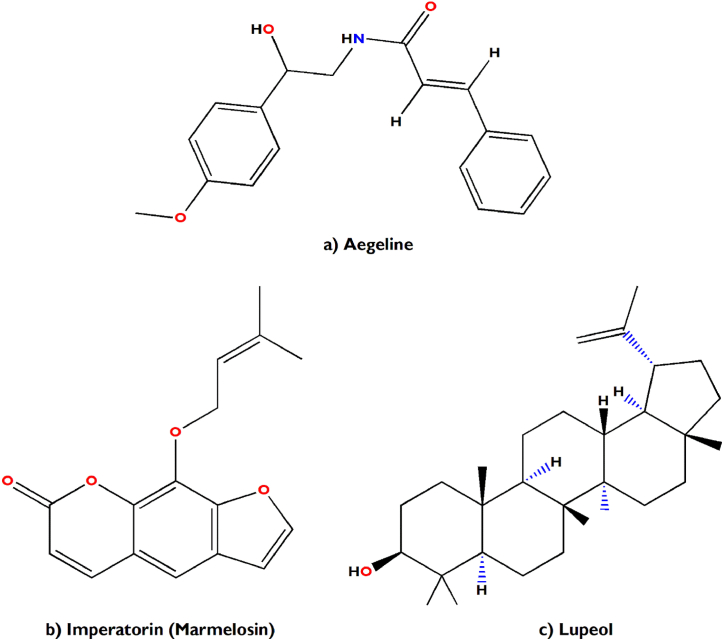
(a–c) Chemical structures of some major anti-indigestion compounds isolated from *Aegle marmelos* Correa.

**Fig. 8:**
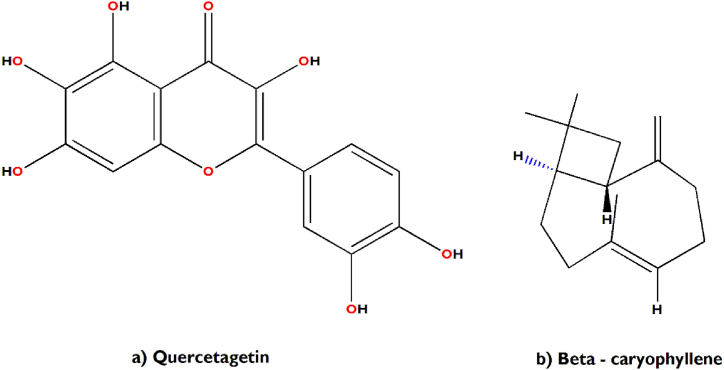
(a–b) Chemical structures of some major wound healing compounds isolated from *Tagetes erecta* L.

**Fig. 9:**
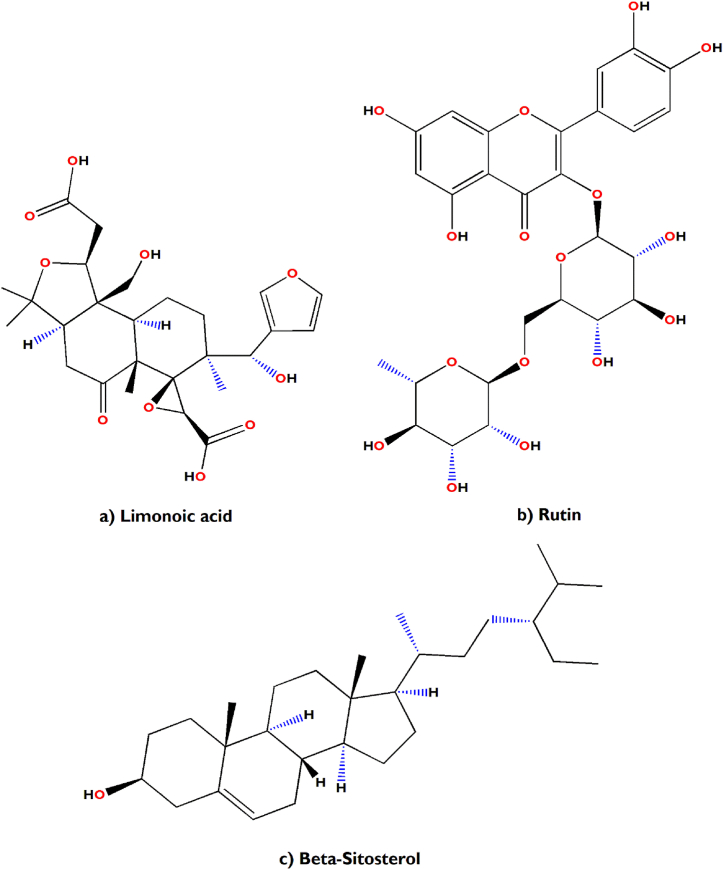
(a–c) Chemical structures of some major antiviral (foot and mouth disease) compounds isolated from *Melia azedarach* L.

**Fig. 10:**
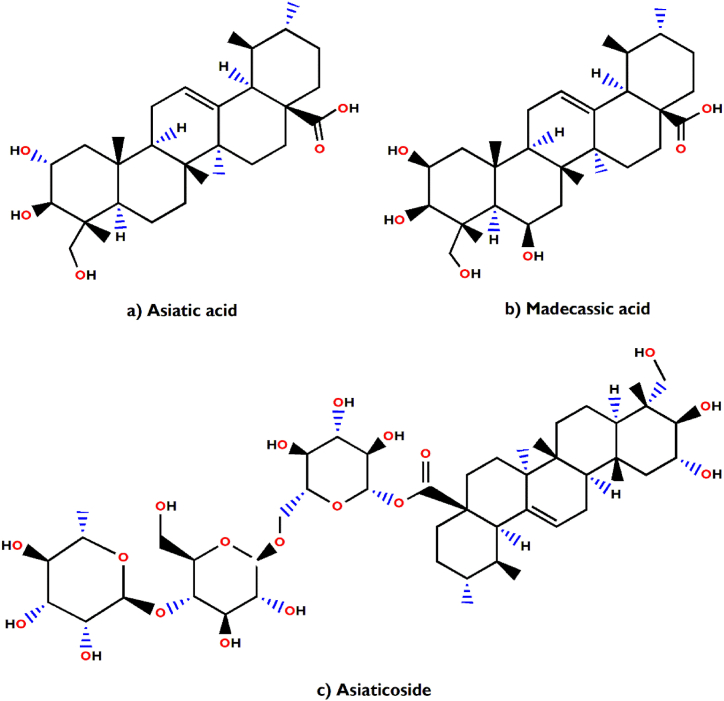
(a–c) Chemical structures of some major neurological disorders healing compounds isolated from *Centella asiatica* (L.) Urb.

**Fig. 11:**
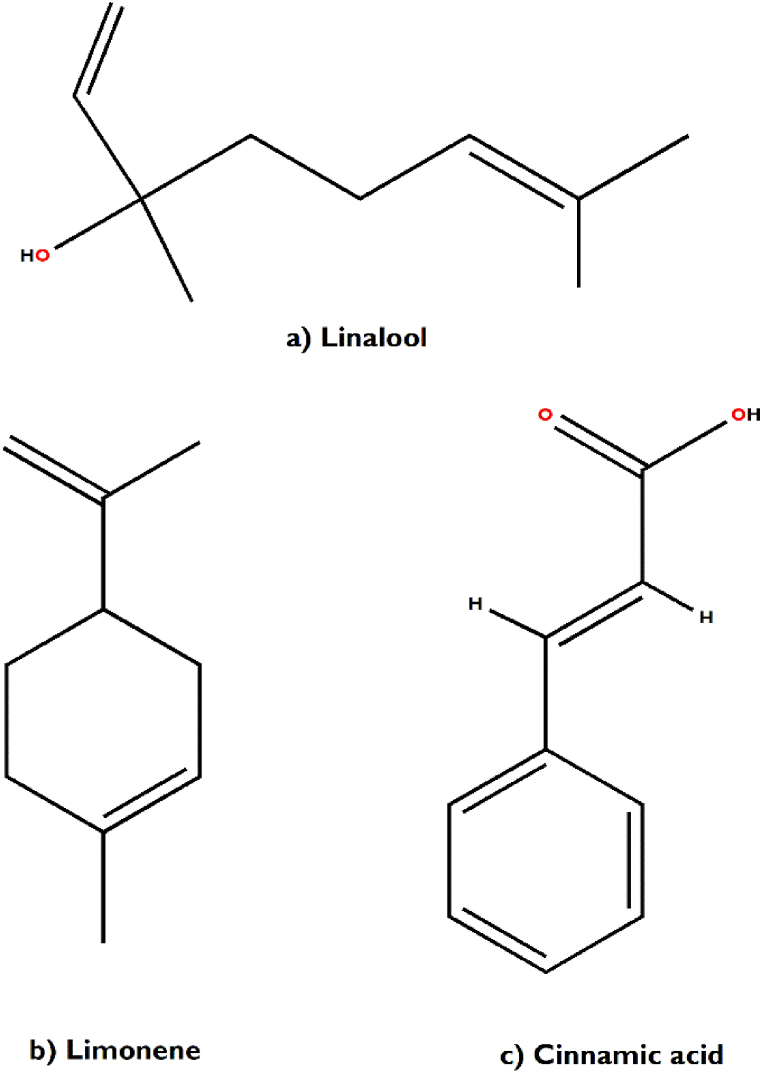
(a–c) Chemical structures of some major deworming healing compounds isolated from *Zanthoxylum armatum* Roxb.

**Fig. 12:**
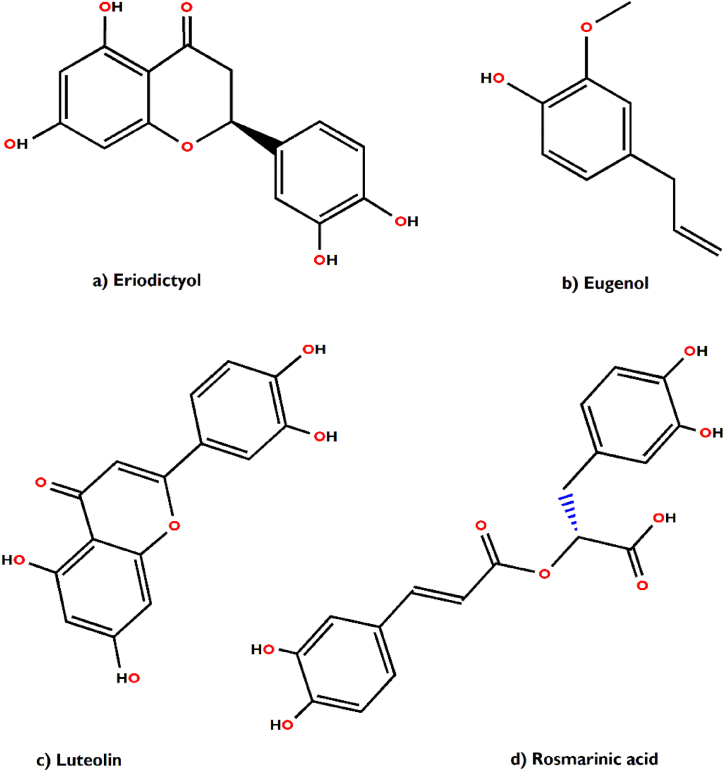
(a–d) Chemical structures of some major poisonous bites healing compounds isolated from *Ocimum basilicum* L.

In support of our study, it has been scientifically proven that *Aegle marmelos* treats Indigestion and also possesses antidiarrheal, antimicrobial and antiviral properties [34]. The bioactive compounds viz., marmelosin, lupeol and aegeline [Fig. 7(a–c)], isolated from *Aegle marmelos* have been reported to be effective against the several bacterial strains (*Bacillus* spp., *Klebsiella aerogenes, Pseudomonas vulgaris, Vibrio cholerae*, *Escherichia coli*, *Shigella* spp., etc.) and showed significant inhibitory action against castor oil induced diarrhoea [152]. This study also indicates that β–caryophyllene and quercetagetin [Fig. 8(a and b)] are a very effective bioactive constituent for the treatment of wound healing, isolated from *Tagetes erecta* [148,153]. β-caryophyllene is a ligand of the cannabinoid receptor 2 (CB2) and on its activation it has the capability to improve wound healing by decreasing inflammation. It also improves re-epithelialization due to enhanced cell proliferation and cell migration [153]. This study also showed that quercetagetin is a very effective in wound healing process in I/R lesions by suppressing MAPK pathway, decreases immune cell infiltration and pro-inflammatory cytokines production [148]. Leaves of *Melia azedarach* used traditionally for foot and mouth diseases has been mostly found to contain meliacine, limonoic acid, β-sitosterol and rutin [Fig. 9(a–c)]. The limonoids of *Melia azeradach* was found to inhibit herpes simplex virus [154] and are cytotoxic against different cancer cell lines [155]. It also indicates that meliacine is significantly suppress the multiplication of foot and mouth disease virus (FMDV) in BHK-21 cells by inhibiting vacuolar acidification [156]. Similarly, β-sitosterol and rutin are involved in the curative properties for inflammation, viral damage, ulcer and immune system booster, by controlling the production of inflammatory cytokines [157,158]. This study also indicates that rutin is an effective constituent against avian influenza strain H5N1 using plaque inhibition assay in the Madin-Darby canine kidney [158]. *Centella asiatica* is another scientifically proven ethnomedicinal plant to have positive effect on diseases of the nervous system by reducing inflammatory factors, ROS production and nerve cell apoptosis, repairing abnormal expression of mitochondrial-related proteins, and improving the survival rate of neural cells [159]. A summary of the related literature showed that the bioactive compounds (asiatic acid, asiaticoside and madecassic acid) isolated from *Centella asiatica* extract [Fig. 10(a–c)] had positive effects against neurological diseases, generally through the mitogen-activated protein kinase (MAPK) signalling pathway by increasing the brain-derived neurotrophic factor (BDNF) contents [160]. The signalling (p38 MAPK and PI3K/Akt/mTOR) pathway can control various events in Alzheimer’s and Parkinson’s disease, viz., neuroinflammation, tau phosphorylation, and synaptic dysfunction [161,162]. The leaves and bark of *Zanthoxylum armatum* containing a major compound of limonene, linalool and cinnamic acid [Fig. 11(a–c)], which shows the highest toxic activity against endoparasites. This study also specifies that limonene is a very effective against *Leishmania* species (*L. major*, *L. braziliensis* and *L. chagasi*) with 50% inhibitory concentrations of 252.0 ± 49.0 and 147.0 ± 46.0 μM, respectively [163]. Similarly, the compounds like linalool, β-ocimene, limonene, α-terpinolene, cinnamone and germacrene isolated from *Z. armatum* leaves exhibits strong DPPH radical scavenging activity (IC50 = 27 μg/mL) relative to activity of ascorbic acid (IC50 = 15.0 μg/mL) [124]. The present study also confirms that *Ocimum basilicum* contains several bioactive constituents like phenols (eugenol), flavonoids (luteolin and eriodictyol) and a polyphenol (rosmarinic acid), which are scientifically studied to have therapeutic properties for venomous bites [Fig. 12(a–d)]. These active compounds isolated from the leaf extract of *O. basilicum* may be the potential mechanism contributing to treat insect stings, snake bites and skin infections [164]. Rosmarinic acid extracted from *O. basilicum* has proved the anti-inflammatory, antioxidant, and neuroprotective activities [165]. Literature also showed that rosmarinic acid has anti-inflammatory properties, stimulates differentiation of cell by controlling the extracellular signal regulating kinase (ERK1/2) signaling pathway and improves cholinergic activities in rat PC12 cells [166]. Rosmarinic acid also prevent Aβ-induced memory loss, inhabits apoptosis and decrease hyperphosphorylation of tau protein [166,167].

## 5 Conclusion

The present study provides trustworthy information on the indigenous ethnoveterinary knowledge of plants. This knowledge is as senescent as human civilization and is transmitted orally from generation to generation. Consequently, it was found that only the older generation is responsible for knowing about native plants and their uses. This knowledge is at risk of being lost due to urbanization, societal changes, and the lack of interest in the uses of plants for curing domestic animals among the younger generation. Even the anthologies of these medicinal plants are unscientific viz., uprooting, scraping bark, etc. which reduces their chances of proliferation.

To overcome these problems, there is an urgent need for collective efforts from taxonomists, ethnobotanists, and pharmacologists to collect, document and conserve this precious folklore knowledge related to the utilization of medicinal and other wild plants. This knowledge is essential for future generations to properly cure their domestic animals through scientific implementation. The inhabitants of the study area need to be aware of sustainable collection, domestication (for personal or trade use), and conservation. This will improve their socio-economic conditions and reduce pressure on natural resources. Lastly, the documented plants require further scientific analysis (phytochemical and pharmacological screening) for their effective utilization for medicinal purposes. In the future, this knowledge could potentially guide the search for developing new pharmacological products.

## Author contribution statement

Nazir Ahmad Bhat: Conceived and designed the experiments; Performed the experiments; Analysed and interpreted the data; Contributed materials, analysis tools or data; Wrote the paper. Licha Jeri, Dolly Karmakar: Performed the experiments; Contributed materials, analysis tools or data; Wrote the paper. Puranjoy Mipun, Nilofer sheikh, Chester John Nongkynrih: Performed the experiments; Contributed materials, analysis tools or data. Pankaj Bharali, Yogendra Kumar: Designed the experiments; Analysed and interpreted the data.

## Additional information

The plant specimens were deposited and preserved in the herbarium of Botany Department, North-Eastern Hill University, Shillong, Meghalaya for future references.

## Declaration of competing interest

The authors declare that they have no known competing financial interests or personal relationships that could have appeared to influence the work reported in this paper.
